# Neuromuscular disease modeling on a chip

**DOI:** 10.1242/dmm.044867

**Published:** 2020-07-07

**Authors:** Jeffrey W. Santoso, Megan L. McCain

**Affiliations:** 1 Laboratory for Living Systems Engineering, Department of Biomedical Engineering, USC Viterbi School of Engineering, University of Southern California, Los Angeles, CA 90089, USA; 2 Department of Stem Cell Biology and Regenerative Medicine, Keck School of Medicine of USC, University of Southern California, Los Angeles, CA 90033, USA

**Keywords:** Skeletal muscle, Motor neurons, Amyotrophic lateral sclerosis, Induced pluripotent stem cells, Tissue engineering, Microfluidic devices

## Abstract

Organs-on-chips are broadly defined as microfabricated surfaces or devices designed to engineer cells into microscale tissues with native-like features and then extract physiologically relevant readouts at scale. Because they are generally compatible with patient-derived cells, these technologies can address many of the human relevance limitations of animal models. As a result, organs-on-chips have emerged as a promising new paradigm for patient-specific disease modeling and drug development. Because neuromuscular diseases span a broad range of rare conditions with diverse etiology and complex pathophysiology, they have been especially challenging to model in animals and thus are well suited for organ-on-chip approaches. In this Review, we first briefly summarize the challenges in neuromuscular disease modeling with animal models. Next, we describe a variety of existing organ-on-chip approaches for neuromuscular tissues, including a survey of cell sources for both muscle and nerve, and two- and three-dimensional neuromuscular tissue-engineering techniques. Although researchers have made tremendous advances in modeling neuromuscular diseases on a chip, the remaining challenges in cell sourcing, cell maturity, tissue assembly and readout capabilities limit their integration into the drug development pipeline today. However, as the field advances, models of healthy and diseased neuromuscular tissues on a chip, coupled with animal models, have vast potential as complementary tools for modeling multiple aspects of neuromuscular diseases and identifying new therapeutic strategies.

## Abstract (precis)

**Summary:** Modeling neuromuscular diseases is challenging due to their complex etiology and pathophysiology. Here, we review the cell sources and tissue-engineering procedures that are being integrated as emerging neuromuscular disease models.

## Introduction

**Fig. 1.**
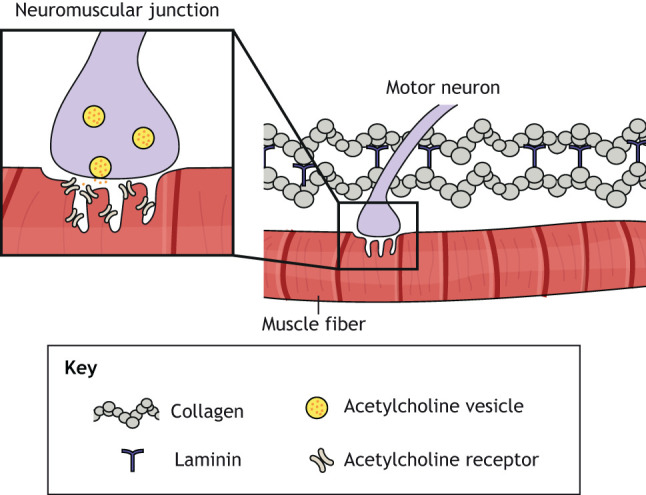
**Schematic of the neuromuscular junction.** Multi-nucleated muscle fibers are innervated by myelinated motor neurons at neuromuscular junctions (NMJs). At the NMJ, motor neurons release acetylcholine vesicles. The neurotransmitter acetylcholine binds to acetylcholine receptors on the membrane of the muscle fiber, causing membrane depolarization and muscle contraction.

Neuromuscular diseases collectively affect 160 per 100,000 people worldwide and are generally characterized by progressive motor impairment and muscular atrophy (Deenen et al., 2015). Although these conditions have diverse etiologies, they each affect one or more components of the motor unit (see Box 1, Fig. 1). For decades, animal models, especially humanized mice (De Giorgio et al., 2019; Nair et al., 2019; Aartsma-Rus and van Putten, 2020), have been the gold standard for neuromuscular disease modeling. More recently, non-mammalian models, such as fruit flies (Lloyd and Taylor, 2010), *Caenorhabditis* *elegans* (Sleigh and Sattelle, 2010) and zebrafish (Babin et al., 2014), have also been used for neuromuscular disease modeling. Although these simpler models are limited by their lower conservation with human genetics, anatomy and physiology compared to mice, they are beneficial because of their lower cost, rapid growth rate, tractable anatomy and ease of genetic manipulation. In general, animal models capture important hallmarks of their human disease counterparts and thus are invaluable for understanding disease progression on an organ- and organism-level scale. However, disease phenotypes in animals can vary widely from humans in terms of progression, severity and other characteristics (De Giorgio et al., 2019; Aartsma-Rus and van Putten, 2020; Babin et al., 2014).

**Box 1. Structure and physiology of the motor unit**

All voluntary movements are controlled by a collection of motor units, each of which comprises a single motor neuron and all the muscle fibers that it innervates (Fig. 1). Motor neurons have a soma that resides in the motor cortex, brain stem or spinal cord, and a single myelinated axon that forms specialized synapses, known as neuromuscular junctions (NMJs), on muscle fibers. Muscle fibers are elongated multi-nucleated cells that are packed with myofibrils, each of which is an interconnected chain of contractile sarcomere units. Multiple muscle fibers are bundled together and wrapped in connective tissue to form a muscle.

Contraction of a motor unit begins when signals from the central nervous system trigger an action potential in the motor neuron, which induces the axon to release the neurotransmitter acetylcholine into the synaptic cleft of the NMJ. Acetylcholine binds to acetylcholine receptors on the membrane of the muscle fiber, which depolarizes the membrane and initiates an action potential. The muscle fiber then propagates this action potential along its length, triggering the entry of extracellular calcium through voltage-sensitive ion channels in the membrane and subsequently a large release of calcium from the sarcoplasmic reticulum. This increase in cytosolic calcium enables the heads of myosin filaments to pull on actin filaments, shortening the sarcomere and ultimately contracting the muscle fiber in an ATP-demanding process. Depending on the frequency of the action potential transmitted by the motor neuron, the muscle fiber undergoes either a singular or sustained contraction, referred to as twitch or tetanus, respectively. Lastly, the free acetylcholine in the NMJ is broken down by acetylcholinesterase, cytosolic calcium is transported back into the sarcoplasmic reticulum, and the membrane potential of the muscle fiber returns to resting levels, thus causing muscle relaxation (reviewed by Hall and Hall, 2015).

Another limitation of animal models is that it is difficult, if not impossible, to recapitulate the genotypic heterogeneity and allelic variation observed in individuals with neuromuscular diseases without generating an unreasonable number of animal strains (Juneja et al., 2019; Morrice et al., 2018). Even monogenic neuromuscular diseases, such as spinal muscular atrophy (SMA), are difficult to model in animals due to patient-specific genotypic features. SMA is an autosomal recessive disease caused by inactivating mutations in the *SMN1* gene, which encodes the survival of motor neuron (SMN) protein (Li, 2017). SMN plays a role in protein homeostasis, cytoskeletal assembly, endocytosis, metabolism and many other processes in motor neurons (Chaytow et al., 2018). SMN shortage or dysfunction causes deficits in axonogenesis, migration, electrophysiology and many other features, leading to neuromuscular junction (NMJ) degeneration and motor neuron death (Laird et al., 2016; McGovern et al., 2015). A second gene, *SMN2*, also produces SMN, but at ∼20% of the levels transcribed from fully functional *SMN1* (Bowerman et al., 2017; Jedrzejowska et al., 2009). SMA has been modeled in mice (Hsieh-Li et al., 2000), *Drosophila* (Spring et al., 2019), zebrafish (McWhorter et al., 2003) and *C. elegans* (Briese et al., 2009) by deleting the endogenous *Smn* gene and overexpressing the human *SMN2* gene. However, the severity and progression of SMA largely depends on the number of *SMN2* copies in a patient (Bowerman et al., 2017; Jedrzejowska et al., 2009), a patient-specific feature of the disease that is nearly impossible to faithfully recapitulate in animals. The only treatment options for SMA are the gene therapy drugs Spinraza (Dangouloff and Servais, 2019) and Zolgensma (Zuroske, 2019), both of which are extremely expensive and thus impractical for many individuals.

Compared to SMA, several neuromuscular diseases have a more heterogeneous genetic etiology, which is even more challenging to model in animals. For example, Charcot-Marie-Tooth (CMT) diseases have been linked to 870 mutations in over 80 genes (McCorquodale et al., 2016), such as *PMP22*, *MPZ*, *GJB1* or *MFN2* (Morena et al., 2019; Saporta et al., 2011). This genetic heterogeneity partially explains the wide range of age of onset and disease symptoms, which usually involve involuntary contraction of limbs and loss of sensation due to axon demyelination. CMT has been modeled in zebrafish and other animal models by introducing a mutation in a single gene known to cause a specific subtype of CMT disease, such as *mfn2* (Chapman et al., 2013) or *prps1* (Pei et al., 2016). However, owing to the vast genetic heterogeneity of CMT diseases, it is infeasible to generate animal models that represent all mutations (Juneja et al., 2019). Largely due to a lack of modular model systems, CMT diseases still lack clinical data supporting any effective treatment beyond physical therapy and pain management (McCorquodale et al., 2016). Mouse models of CMT have also demonstrated that impaired development of the NMJ precedes synaptic deficits (Sleigh et al., 2013; Spaulding et al., 2016), suggesting that microscale models of the motor unit might be useful for elucidating the pathophysiology of this broad group of diseases.

Amyotrophic lateral sclerosis (ALS) is another neuromuscular disease that introduces unique challenges for modeling in animals because it can be either inherited (10%) or sporadic (90%) (Boylan, 2015). In ALS, over 50 genes either directly cause motor neuron death or alter key functions, such as vesicle trafficking, axonal structure and cytoskeletal stability (Boylan, 2015; Seminary et al., 2018; Shi et al., 2018). The most commonly affected genes are *C9ORF72*, *SOD1*, *TARDBP* and *FUS*, usually occurring in some kind of combination (Lattante et al., 2015; Nguyen et al., 2018). Animal models of ALS have been generated by expressing a mutated version of one of these human genes in mice (Ripps et al., 1995; Zhang et al., 1997; Devoy et al., 2017), *Drosophila* (Şahin et al., 2017; Watson et al., 2008; Perry et al., 2017; Xu et al., 2013), zebrafish (Shaw et al., 2018; Lissouba et al., 2018) and *C. elegans* (Oeda et al., 2001; Wang et al., 2009). Additionally, environmental factors, such as pesticides, flame retardants and military-related trauma, have been correlated to ALS (Su et al., 2016). However, the small number of clinical cases and limited model systems make assigning causality from environmental factors very difficult. The natural process of aging has also been tied to ALS, probably because of the aggregation of misfolded proteins and oxidative stress (Jang and Van Remmen, 2011; Turner et al., 2012). Owing, in large part, to the many causes and complex pathophysiologies of ALS, the only therapies are the anti-glutamatergic compound riluzole and the antioxidant edaravone, both of which only assuage symptoms and extend survival for a few months (Nguyen et al., 2018).

Neuromuscular diseases can also be caused by factors external to the motor unit. For example, myasthenia gravis (MG) is a sporadic autoimmune disease in which auto-antibodies selectively destroy acetylcholine receptors, causing a reduction in NMJ signal transmission (Phillips and Vincent, 2016). A mouse model of MG has been developed by injecting rat acetylcholine receptors into mice, which then triggered the development of auto-antibodies to their own acetylcholine receptors (Granato et al., 1976). However, MG cannot be modeled in simple organisms such as *Drosophila* and *C. elegans* because they lack an adaptive immune system. The causes of MG are still mostly unknown and treatment is limited to acetylcholinesterase inhibitors, immunosuppressants or a thymectomy (Gilhus, 2016), highlighting the need for additional predictive model systems to develop more targeted therapies.

Collectively, these examples highlight that neuromuscular diseases are very diverse and are characterized by many complex genetic and non-genetic etiologies and pathophysiologies. These complexities introduce many challenges for developing comprehensive animal models. Thus, new disease models that are more efficient and predictive are essential for accelerating our mechanistic knowledge of these diseases, as well as the discovery of effective therapies. Integrating patient-derived cells with microfabricated *in vitro* platforms, known as organs-on-chips, is an emerging solution to fill the gaps of animal models and holds promise for patient-specific neuromuscular disease modeling and drug development. As discussed below, these platforms are often developed using animal cells or cell lines that are easy to scale, and that can provide important proof-of-concept and basic physiological information. To address issues of human relevance, animal cells or cell lines can then be replaced with patient-derived cells, which can be acquired from a variety of sources. In the next section, we describe the cell sources for neuromuscular disease models, which can ultimately be integrated into the two- (2D) and three-dimensional (3D) engineered tissue platforms described in the following sections.

## Cell sources for *in vitro* models of neuromuscular tissues

*In vitro* models can mitigate many of the limitations of animal models described above, such as human relevance and scalability. However, the usefulness of any *in vitro* model is highly dependent on the source and structural and functional maturity of its cells. This is especially complex when modeling neuromuscular tissues, which consist of both muscle cells and motor neurons. In this section, we will describe the types of muscle and motor neuron cell types available today for *in vitro* models, and weigh up their advantages and disadvantages.

### Skeletal muscle cells

**Fig. 2.**
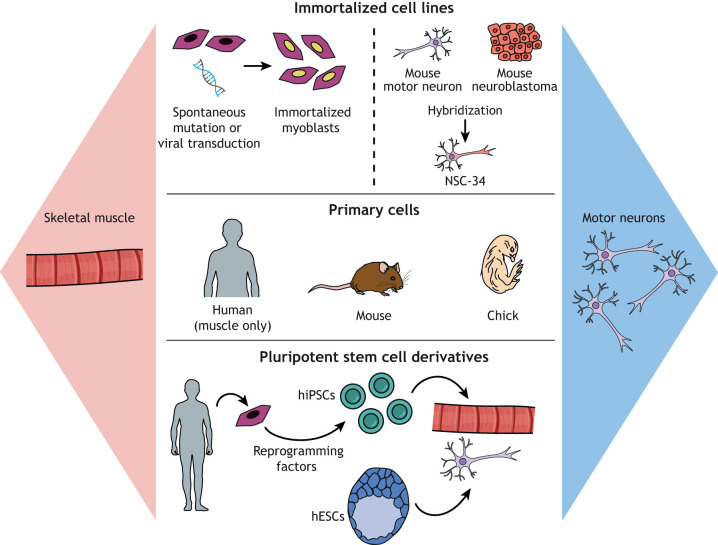
**Muscle and motor neuron cell sources for *in vitro* models.** Myoblasts and motor neurons available for *in vitro* models fall into three categories: immortalized cell lines, primary cells and pluripotent stem cell derivatives. Immortalized cell lines recapitulate the basic properties of the original cell type and are inexpensive and easy to expand in culture. However, the immortalization process causes de-differentiation and a loss of important structural and functional features. Primary cells are usually the most mature and physiologically relevant cell source but are relatively costly and difficult to obtain, and have a limited ability to expand in culture. Pluripotent stem cells can be widely expanded in culture and then differentiated into myoblasts or motor neurons. Induced pluripotent stem cells have the additional advantage of patient specificity. However, pluripotent stem cell derivatives are generally heterogeneous and immature. hESC, human embryonic stem cell; hiPSC, human induced pluripotent stem cell.

Generating skeletal muscle tissue *in vitro* is a multi-step process. First, mononuclear skeletal myoblasts are seeded on a standard culture surface that is often coated with extracellular matrix (ECM) proteins, such as collagen or laminin. The myoblasts are then expanded in a high-serum growth medium until they reach confluence. The medium is then substituted with a low-serum differentiation medium that triggers the fusion of myoblasts into multi-nucleated myotubes, the *in vitro* surrogate to muscle fibers (Neville et al., 1997). As illustrated in Fig. 2, several different sources of myoblasts are currently available, each with distinct advantages and disadvantages that are important to consider when engineering neuromuscular disease models.

Primary myoblasts are harvested from embryonic or adult animals, such as chicks (Urja et al., 2018; Vallette et al., 1986) or mice (Hindi et al., 2017), by excising muscle tissue and either enzymatically digesting it to a cell suspension or collecting the cells that migrate from cultured tissue explants (Vaughan and Lamia, 2019). Myoblasts are then purified using simple pre-plating steps or more sophisticated techniques, such as magnetic cell sorting (Sincennes et al., 2017; Spinazzola and Gussoni, 2017). Human primary myoblasts can be isolated from muscle tissue collected during a surgical procedure or by needle biopsy (Joyce et al., 2012), and similarly processed and purified.

The structural and functional properties of myotubes differentiated from primary myoblasts closely recapitulate those of native muscle, such as a high density of myofibrils and spontaneous contractile behavior (Pimentel et al., 2017). However, primary myoblasts can only be passaged a few times before their growth rate and myogenic capacity decline, leading to limited supply and passage-dependent variability. These supply and variability issues are especially problematic for human primary myoblasts, which are generally isolated from relatively small muscle biopsies. Moreover, primary myoblasts can vary widely in purity and functional maturity depending on the isolation and purification methods, and the characteristics of the subject (Cheng et al., 2014; Soriano-Arroquia et al., 2017). This issue is further exacerbated by data indicating a beneficial role for fibroblasts in myotube function (Rao et al., 2013), raising questions about the ideal purity of primary myoblasts for *in vitro* studies and adding further variability to the performance of myotubes differentiated from primary myoblasts.

Compared to primary myoblasts, immortalized myoblast cell lines are a more convenient source of cells that are relatively pure and easy to expand. The most common myoblast cell line is C2C12, which was isolated from 2-month-old mouse muscle in 1977 (Yaffe and Saxel, 1977). Another common cell line is L6, which was isolated from newborn rat muscle in 1968 (Yaffe, 1968). Both C2C12 and L6 cells proliferate rapidly with a generation time of ∼24 h and fuse into multi-nucleated contractile myotubes (McMahon et al., 1994; Öberg et al., 2011). Thus, they are a convenient model system for investigating processes such as myotube fusion (Zhao et al., 2015) or degeneration (Menconi et al., 2008). These cells are also compatible with gene transfection (Balcı and Dinçer, 2009), which is useful for establishing the functions of genetic variants in myoblast growth or fusion (Prinsen and Veerkamp, 1998), or introducing disease-relevant mutations (Liang et al., 2016).

Myotubes differentiated from cell lines have lower levels of structural and functional maturity, and distinct metabolic properties compared to myotubes differentiated from primary myoblasts (Abdelmoez et al., 2019; Robinson et al., 2019), probably because the immortalization process causes some amount of de-differentiation and loss of myogenic properties that continue to decline with increasing passage number. Thus, although myoblast cell lines are a reproducible and cost-effective cell source compared to primary myoblasts, myotubes differentiated from cell lines have limited relevance to native muscle tissue. Furthermore, a commercialized human myoblast cell line does not currently exist, raising further concerns about the translatability of data collected using common myoblast cell lines, which are derived from rodents. However, primary human myoblasts have been immortalized by the forced expression of a telomerase subunit and cyclin dependent kinase 4, which blocks a stress pathway (Mamchaoui et al., 2011). Myotubes generated from these cell lines produce relatively mature sarcomeres (Morris et al., 2020) and form NMJs when co-cultured with motor neurons (Saini et al., 2019), suggesting that they could become a promising cell source.

To overcome the limitations of primary myoblasts and myoblast cell lines, several protocols for deriving myogenic cells from human embryonic stem cells (hESCs) or human induced pluripotent stem cells (hiPSCs) have recently emerged (Salani et al., 2012). These cells have the advantages of human origin and essentially limitless supply, as hESCs and hiPSCs can be expanded in culture for many passages without loss of functionality. hESCs and hiPSCs are also compatible with gene editing techniques, such as CRISPR/Cas9, which can be used to introduce or correct select disease-relevant mutations (Shi et al., 2018; Young et al., 2016). Because hiPSCs are reprogrammed from somatic cells, such as skin fibroblasts, hiPSC-derived myogenic cells can be used to generate patient-specific myotubes, which makes them especially desirable for modeling inherited neuromuscular diseases.

One approach for generating myogenic progenitors from hiPSCs is to overexpress master regulators of myogenic differentiation, such as *MYOD1* (Abujarour et al., 2014; Rao et al., 2018) or *PAX7* (Darabi et al., 2012). A similar reprogramming process has also been used to directly transdifferentiate other cell types, such as fibroblasts, into myogenic progenitors (Boularaoui et al., 2018; Ito et al., 2017; Lattanzi et al., 1998). A second approach, known as directed differentiation, guides hiPSCs through native-like myogenic developmental pathways by sequentially adding small molecules that activate or suppress specific signaling pathways (Chal et al., 2016, 2015; Maffioletti et al., 2015; Shelton et al., 2016; van der Wal et al., 2018; Xi et al., 2017). Directed differentiation is generally slower than transdifferentiation, but the resulting myogenic progenitors are thought to be a closer match to native myoblasts because they follow a more natural differentiation process (Jiwlawat et al., 2018).

Although impressive progress has been made in deriving myogenic progenitors from hiPSCs, most current protocols generally suffer from wide variability and low efficiency (Jiwlawat et al., 2018). These issues limit cell yield and purity. However, protocols for cryopreserving and expanding hiPSC-derived myogenic progenitors are being developed (van der Wal et al., 2018), which helps mitigate issues with differentiation variability and throughput. Despite these practical limitations related to cell differentiation, hESC- and hiPSC-derived myogenic progenitors successfully fuse into myotubes that contain myofibrils and exhibit key functional behaviors, such as calcium cycling and contractility (Rao et al., 2018; Skoglund et al., 2014). However, myofibrils in hESC- and hiPSC-derived myotubes still have immature features compared to native muscle fibers or myotubes derived from primary myoblasts (Lainé et al., 2018).

The structural and functional immaturity of myotubes probably contributes to the stunted maturation of NMJs that form between hiPSC-derived myotubes and motor neurons. However, several approaches for maturing hiPSC-derived myotubes are under development, such as identifying small molecules that boost maturation (Selvaraj et al., 2019) or applying other strategies discussed below. Additionally, the maturation of C2C12 myotubes has been improved by applying biophysical cues, such as mechanical stretch (Chang et al., 2016; Heher et al., 2015) or electrical stimulation (Ito et al., 2014; Nedachi et al., 2008), which might have similar benefits for hiPSC-derived myotubes. Thus, although hiPSC-derived myotubes have significant potential as an essentially limitless source of patient-specific myotubes, researchers need to enhance the differentiation efficiency and maturity of these cells to improve their throughput and relevance for modeling neuromuscular diseases *in vitro*.

### Motor neurons

Compared to myoblasts, fewer sources of motor neurons exist for *in vitro* models. Because most motor neurons stem from the spinal cord and project onto muscle fibers, it is not possible to isolate intact primary motor neurons from humans. However, primary motor neurons can be isolated from embryonic or adult mice by extracting and digesting spinal cord tissue and using density gradient separation to isolate motor neurons from supporting cell types, such as astrocytes and other glial cells (Beaudet et al., 2015; Gingras et al., 2007). Although rodents are a viable source of primary motor neurons, these cells are not human, which limits their relevance for neuromuscular disease modeling. Furthermore, the cell yield is relatively low and cannot be increased with passaging because motor neurons are terminally differentiated and non-proliferative.

Because motor neurons do not proliferate, true motor neuron cell lines do not exist. However, a hybrid mouse cell line (NSC-34) has been generated by fusing neuroblastoma cells with embryonic motor neurons. NSC-34 cells retain the proliferative properties of the tumor cells while also exhibiting select neuronal properties, such as acetylcholine synthesis, neurotransmitter release and neurofilament proteins (Cashman et al., 1992). This cell line has been used to measure the neurotoxicity of drugs (Maier et al., 2013) and receptor trafficking (Matusica et al., 2008), and has also been transfected to introduce mutations relevant to ALS (Gomes et al., 2010; Pinto et al., 2017). Similar to myoblast cell lines, the disadvantage of these cells is their non-human origin and limited relevance to native motor neurons. For example, these cells do not replicate glutamate-mediated excitotoxicity (Madji Hounoum et al., 2016), questioning their ability to replicate key features of neuromuscular diseases.

Human motor neurons can also be derived from hESCs and hiPSCs via reprogramming or directed differentiation. hESCs and hiPSCs have been reprogrammed into motor neurons by overexpressing *NGN2* (also known as *NEUROG2*), *ISL1* and *LHX3* (Goto et al., 2017; Hester et al., 2011; Lee et al., 2012). Human fibroblasts have also been transdifferentiated into motor neurons by overexpressing eight genes (Son et al., 2011). Several directed differentiation methods have also been established, which entail dosing hESCs or hiPSCs with a combination of neurotrophic factors, retinoic acid, sonic hedgehog and Notch inhibitors (Du et al., 2015; Hu and Zhang, 2009; Li et al., 2008b; Qu et al., 2014; Shimojo et al., 2015). Motor neurons have also been differentiated from the human fetal spinal cord stem cell line NSI-566RSC (Guo et al., 2010), which serves as another relatively accessible source of human motor neurons.

Similar to other stem cell derivatives, stem cell-derived motor neurons can be limited by cell heterogeneity, varying differentiation efficiency and stunted maturation (Ichida et al., 2018). Although mouse motor neurons derived from transdifferentiated fibroblasts or directly differentiated iPSCs have a transcriptome that is similar to primary motor neurons (Ichida et al., 2018), how the structural and functional properties of these cells compare to their primary counterpart is mostly unknown. Despite the limited functional characterization of these cells, hiPSC-derived motor neurons have already been shown to be a promising cell source for patient-specific modeling of neuromuscular diseases such as ALS (Dimos et al., 2008; Sances et al., 2016; Sareen et al., 2013; Shi et al., 2018) and SMA (Fuller et al., 2016; Murdocca et al., 2016). Thus, hiPSC-derived motor neurons are likely to contribute to the development of new therapies for these diseases that account for the genotype of the patient*.*

## Engineered *in vitro* models of neuromuscular tissues

In addition to cell source, another important consideration for *in vitro* model development is the configuration of the cells, such that the cultured tissue is anatomically relevant and integrated with assays to measure functional phenotypes. Initial approaches for engineering neuromuscular tissues *in vitro* entailed simply seeding dissociated motor neurons (Daniels et al., 2000; Das et al., 2010; Guo et al., 2011; Kengaku et al., 1991; Son et al., 2011; Umbach et al., 2012) or spinal cord explants (Askanas et al., 1987; Braun et al., 1997) on top of a 2D layer of myotubes attached to a conventional culture surface. Over the course of several days, the neurons extend axons and form NMJs with the myotubes that successfully exhibit functional post-synaptic potentials. However, image analysis has revealed blotchy colocalization of pre- and post-synaptic markers and poor acetylcholine receptor clustering in these simple co-cultures compared to native NMJs (Das et al., 2010; Umbach et al., 2012). This limited synaptic maturity brings into question the ability of these culture systems to accurately model disease-relevant phenotypes.

The relatively stunted NMJ development in conventional co-cultures could be attributed to many factors. First, without spatial organization cues, myoblasts fuse into branched myotubes with random orientations that poorly recapitulate the architecture of native muscle fibers (Bettadapur et al., 2016; Denes et al., 2019), which can limit the formation of elongated myofibrils and mature sarcomeres. Second, myotubes often delaminate from conventional culture surfaces within ∼2 weeks as they generate increasing amounts of mechanical stress (Wang et al., 2012; Sun et al., 2013). This can probably be attributed to both the high stiffness of conventional culture substrates and the limited number of cell-adhesive molecules presented on their surfaces (Bettadapur et al., 2016). Limited culture lifetime is especially problematic for engineering neuromuscular tissues because NMJ maturation probably requires longer than 2 weeks. Third, *in situ*, motor neuron soma are located in the spinal cord and only the axons of motor neurons physically interact with muscle fibers. Thus, seeding motor neurons on top of myotubes is not anatomically relevant and might alter the physiology of one or both cell types.

Simple mixed co-cultures also suffer from technical problems that limit data collection. For example, measuring forces generated by cells is not possible on most, if not all, conventional culture substrates, precluding quantitative assessment of muscle force production due to motor neuron stimulation. This is a key functional readout in animal models, with high relevance to the severity of neuromuscular disease (Bonetto et al., 2015). A second limitation is that mixed co-cultures afford minimal independent control or analysis of each cell type, as the cells are cultured in the same medium and any electrical stimulation or drug treatment reaches both cell types simultaneously. Similarly, isolating material from each cell type independently to measure changes in gene or protein expression, which is often important for establishing disease mechanisms as well as drug effects, is challenging.

To overcome these diverse biological and technical challenges, researchers have developed several types of tunable culture surfaces and microfabricated devices to engineer more sophisticated neuromuscular tissues *in vitro*. These surfaces and devices are usually also integrated with assays for quantifying structural and functional tissue phenotypes. In particular, when coupled with the hiPSC-derived cell sources described above, these platforms, known as organs-on-chips or microphysiological systems, have immense potential for advancing neuromuscular disease modeling and drug development. In this section, we describe both 2D and 3D engineered tissue models.

### Engineered 2D models of neuromuscular tissues

Engineering a neuromuscular tissue *in vitro* depends on first culturing mature and stable myotubes. In native skeletal muscle fibers, the ECM plays a key role in tissue development and physiology by binding to integrin receptors and providing biomechanical support as the muscle fibers contract (Gillies and Lieber, 2011). The ECM is also a rich source of biochemical cues that regulate behaviors such as adhesion, proliferation and differentiation. Furthermore, the ECM plays an active role in skeletal muscle disease, injury and aging, with fibrosis and subsequent tissue stiffening contributing to diminished muscle function (Mann et al., 2011). Owing to the documented importance of the ECM in native muscle fibers, several types of tunable culture substrates that mimic aspects of native muscle ECM have been developed, as described below. However, another important feature of *in vitro* models of neuromuscular tissues is the ability to measure muscle contractility in response to motor neuron stimulation. Thus, we also describe later in this section how engineered substrates have been integrated with contractility assays.

#### Natural biomaterial substrates

Hydrogels synthesized from natural polymers are popular culture substrates due to their biocompatibility, although they can suffer from batch-to-batch variability. Collagen hydrogels are routinely used as culture substrates for myoblasts and myotubes due to their intrinsic bioactivity (Palade et al., 2019), as we also discuss above. Aligned myotubes have been fabricated on collagen hydrogels by embedding topographical features into the hydrogel (Kim et al., 2017) or using ultrasound to pattern the cells acoustically (Armstrong et al., 2018). Gelatin, a partially hydrolyzed form of collagen, is also crosslinked into thermostable hydrogels by either mixing gelatin polymers with enzymatic crosslinking agents (Bettadapur et al., 2016; Denes et al., 2019; Suh et al., 2017) or methacrylating gelatin polymers such that they are compatible with photopolymerization techniques (Hosseini et al., 2012; Sun et al., 2018). Crosslinking increases the stiffness of the hydrogel and reduces its degradability (Sun et al., 2018), which can be advantageous for *in vitro* neuromuscular models that need to be stable for several weeks.

To promote myotube alignment on gelatin hydrogels, the surface can be micromolded with polydimethylsiloxane (PDMS) (a silicone elastomer) stamps with ridges several micrometers in size (Bettadapur et al., 2016; Chal et al., 2016; Denes et al., 2019; Hosseini et al., 2012). PDMS stamps are fabricated by casting PDMS on silicon wafer templates made using photolithography, which can generate feature sizes of ∼1 µm (Suh et al., 2017). Probably due to their enhanced bioactivity, micromolded gelatin hydrogels can extend the culture lifetime and maturation of C2C12 myotubes compared to synthetic culture surfaces (Bettadapur et al., 2016; Denes et al., 2019). Carbon nanotubes have also been embedded into methacrylated gelatin hydrogels to enhance myotube maturation by increasing electrical conductivity (Ahadian et al., 2015; Ramón-Azcón et al., 2013). To better mimic the basement membrane of muscle, micromolded gelatin hydrogels have also been crosslinked with a layer of laminin, which improves the adherence, morphology and electrophysiology of myotubes and neural cells (Besser et al., 2020).

#### Synthetic biomaterial substrates

Synthetic biomaterials are advantageous culture substrates because their mechanical and biochemical properties are highly controllable and reproducible. For example, polyethylene glycol and polyacrylamide (PA) are both biologically inert hydrophilic polymers that can be crosslinked into hydrogels with elastic moduli tuned to match the developing, healthy or fibrotic muscle tissue matrices (Engler et al., 2004). Another synthetic biomaterial that is implemented as a culture surface is the aforementioned PDMS. The elasticity of PDMS can be easily tuned to physiological or pathological values by altering the ratio of base to crosslinker (Wang et al., 2014) or blending different formulations of PDMS (Palchesko et al., 2012).

To achieve consistent cell adhesion, researchers must functionalize the synthetic substrate with ECM proteins. This is generally considered an advantage because ECM ligand type and concentration can be specified. Because collagen accounts for up to 10% of the dry weight of muscle (Gillies and Lieber, 2011), several studies have fabricated substrates for C2C12 cultures by transferring collagen onto PDMS (Duffy et al., 2016) or PA hydrogels (Engler et al., 2004; Li et al., 2008a). Because the basement membrane of muscle fibers is enriched in laminin and fibronectin, synthetic substrates functionalized with either of these glycoproteins also promote myoblast adhesion and fusion into myotubes (Duffy et al., 2016; Gilbert et al., 2010; Palchesko et al., 2012; Ziemkiewicz et al., 2018).

**Fig. 3.**
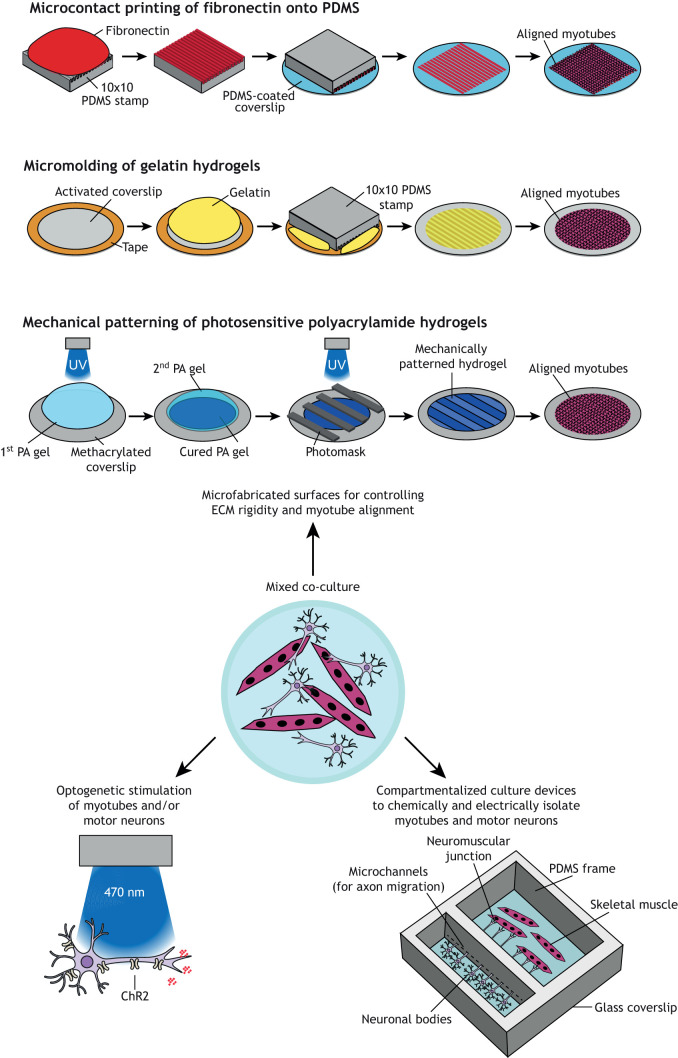
**Engineered** **2D** **neuromuscular** **tissues.** Conventional approaches for engineering neuromuscular tissues *in vitro* entailed mixed co-cultures (center). New advances to improve the architecture and assaying capabilities of 2D neuromuscular tissues include microfabricated surfaces (top), compartmentalized culture devices (right), and integration of optogenetics (left). ChR2, channelrhodopsin-2; ECM, extracellular matrix; PA, polyacrylamide; PDMS, polydimethylsiloxane; UV, ultraviolet.

Synthetic biomaterials are also compatible with many micropatterning techniques that can be used to introduce microscale features on the surface to spatially control cell adhesion and alignment (Falconnet et al., 2006), as shown in Fig. 3. For example, PDMS stamps generated using the same photolithography techniques described above can be used to transfer ECM proteins onto a surface in a process known as microcontact printing (Qin et al., 2010). This process has been used to prescribe myotube alignment on Petri dishes (Bajaj et al., 2011), PDMS-coated surfaces (Bettadapur et al., 2016; Jiwlawat et al., 2019; Nesmith et al., 2016; Palchesko et al., 2012; Sun et al., 2013) and PA hydrogels (Li et al., 2008a). Photolithography has also been used to selectively expose strips of a PA hydrogel to UV light, which activates only the exposed regions for collagen binding and thus myoblast adhesion (Engler et al., 2004). Myotubes have also been aligned on substrates with nanoscale ridges fabricated using electron beam lithography, in which electrons are scanned in a defined pattern on a wafer coated with a light-sensitive photoresist (Wang et al., 2012). Another alternative is solvent-assisted capillary force lithography, in which a polymer solution is molded on a silicon wafer with features at the hundreds of nanometers scale (Yang et al., 2014).

#### Integrated contractility assays

Micropatterned synthetic culture substrates are especially compatible with assays that quantify myotube contractility because the mechanical properties of the substrate are well defined, and myotube architecture can be controlled to increase the magnitude and reproducibility of contractile force production. Micropatterned PA gels are widely used as a substrate for traction force microscopy, a technique that quantifies forces generated by cells by tracking the displacement of fluorescent beads embedded in the hydrogel. Although traction force microscopy is more commonly used for cardiac myocytes (Ariyasinghe et al., 2017; McCain et al., 2012; Pasqualini et al., 2018; Ribeiro et al., 2015), it has also been used to quantify forces generated by micropatterned C2C12 myotubes (Li et al., 2008a).

Contractility can also be quantified by culturing myotubes on flexible cantilevers, as in the muscular thin film (MTF) assay. The MTF assay entails first spin coating a glass coverslip with a layer of poly(N-isopropylacrylamide) (PNIPAAm), a temperature-sensitive polymer, followed by a layer of PDMS (Feinberg et al., 2007). The PDMS is then laser-cut into arrays of cantilevers with dimensions ranging from 1 mm to 5 mm (Agarwal et al., 2013), microcontact printed with lines of fibronectin, and used to culture myotubes. After the desired culture period, the muscle-PDMS cantilevers, referred to as MTFs, are released by reducing the temperature from 37°C to 25°C to solubilize the PNIPAAm. Electrodes are then used to stimulate myotube contraction, which causes cantilever bending. Contractile stress is calculated based on the radius of curvature of each MTF (Grosberg et al., 2011). The MTF assay has been successfully used to measure twitch and tetanus forces generated by C2C12 myotubes (Sun et al., 2013) and primary human myotubes (Nesmith et al., 2016). Microfabricated silicon cantilevers with dimensions of <1 mm have also been used as a culture substrate for primary rat myotubes, and the contractile stresses in this system are measured based on the myotube-induced deflection of laser light (Smith et al., 2014a; Wilson et al., 2010). These compact laser systems are advantageous for multiplexing, which increases testing throughput and scalability for drug screening applications (Smith et al., 2014b).

#### Engineered co-cultures of skeletal muscle and motor neurons

Microfabricated surfaces have also been developed to improve mixed co-cultures of myotubes and motor neurons. For example, photopolymerization techniques have been used to fabricate PA hydrogels with alternating soft and stiff stripes that mimic the rigidity of nervous tissue and muscle tissue, respectively (Happe et al., 2017). On these surfaces, myoblasts preferentially migrate onto the stiffer stripes and fuse into aligned myotubes. When co-cultured with motor neurons, myotubes on mechanically patterned hydrogels exhibited increased acetylcholine receptor clustering compared to myotubes co-cultured on uniform hydrogels (Happe et al., 2017).

NMJ maturation has also been achieved in mixed co-cultures by applying electrical stimulation using a bioreactor (Charoensook et al., 2017). This approach could be further refined by transfecting one or both cell types with channelrhodopsin, a membrane channel that is activated by blue light (Fig. 3). Because chronic optogenetic stimulation of myotubes can improve maturity (Rangarajan et al., 2014), a similar strategy applied to co-cultures could be a relatively non-invasive approach for maturation. Transfecting motor neurons with channelrhodopsin is also a powerful experimental tool for mixed co-cultures because it enables users to stimulate only motor neurons and therefore more clearly identify responses in the muscle that are driven specifically by motor neurons (Lin et al., 2019; Steinbeck et al., 2016).

Compartmentalized culture devices have also been microfabricated to physically isolate motor neurons and myotubes into separate chambers (Fig. 3). These chambers are connected by microchannels that are permissive to axons, but not cell bodies, to allow the controlled formation of NMJs in the myotube chamber (Santhanam et al., 2018; Taylor et al., 2003). Because the chambers are chemically isolated, these devices allow each cell type to be cultured in its own medium, which may boost viability. Furthermore, drugs or other small molecules can be selectively added to one or both chambers (Santhanam et al., 2018), which can be useful for establishing drug mechanisms. Structural and functional analyses are also easier in these devices compared to mixed co-cultures because NMJs form in relatively prescribed locations and cells in each chamber can be electrically stimulated independently.

### Engineered 3D models of neuromuscular tissues

**Fig. 4.**
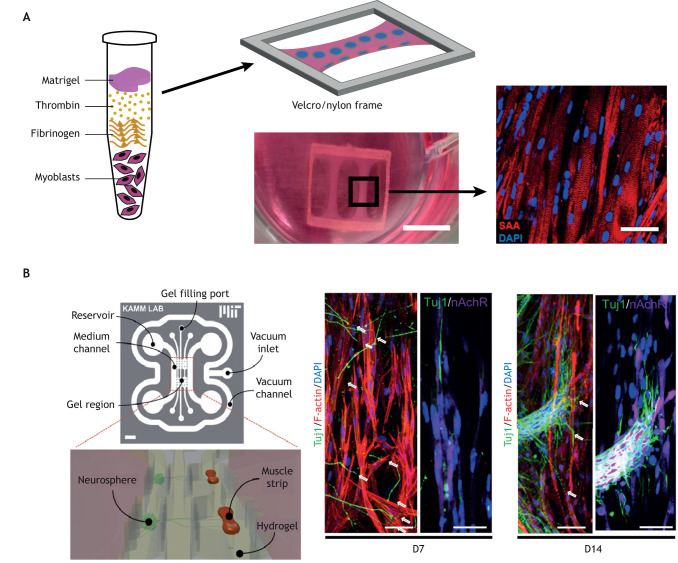
**Engineered** **3D** **neuromuscular** **tissues.** (A) Aligned 3D muscle bundles are engineered by mixing myoblasts in an ECM pre-polymer solution of Matrigel, thrombin and fibrinogen, and casting it into a microfabricated support structure, such as a Velcro frame. Scale bars: 50 mm (left image); 50 µm (right image). Adapted from Madden et al. (2015). (B) Compartmentalized fluidic devices have been microfabricated to controllably co-culture 3D muscle bundles and motor neuron spheroids, and generated NMJs after 14 days in culture. D7, day 7; D14, day 14; DAPI, 4′,6-diamidino-2-phenylindole; nAChR, nicotinic acetylcholine receptor; SAA, sarcomeric alpha-actinin; Tuj1, neuron-specific class III β-tubulin. Scale bars: 2 mm (left image); 10 µm (right image). Adapted with permission from Osaki et al. (2018) and Uzel et al. (2016). The images in this figure are not published under the terms of the CC-BY license of this article. For permission to reuse, please see Madden et al. (2015), Osaki et al. (2018) and Uzel et al. (2016).

Although engineered 2D neuromuscular tissues have many advantages from an assay perspective, they fundamentally lack the bundle-like architecture and cell-ECM interactions of native muscle fibers. To address this, researchers have developed several approaches to engineer miniature 3D muscle bundles (Fig. 4). These types of approaches were first reported in the late 1990s and entailed injecting primary myoblasts mixed in an ECM pre-polymer solution into a rectangular chamber with patches of stainless-steel screening (Shansky et al., 1997) or Velcro (Powell et al., 1999) at its longitudinal ends. As the myoblasts fused into myotubes, they detached from the bottom surface but remained embedded in the ECM and anchored by the screening or Velcro, forming an elongated 3D muscle bundle with aligned myotubes.

Over the past two decades, approaches for engineering 3D muscle bundles have been advanced and refined. Several different types of culture chambers with anchor points have been fabricated (Costantini et al., 2017a; Smith et al., 2016), including Velcro and nylon frames (Davis et al., 2017, 2019; Madden et al., 2015; Rao et al., 2018; Smith et al., 2016; Zhang et al., 2018) and microfabricated chambers with pillars (Osaki et al., 2018, 2020; Uzel et al., 2016). Testing of multiple ECM solutions has also revealed that fibrin hydrogels are optimal for encapsulating myotubes due to their strength (Hinds et al., 2011; Pollot et al., 2018), although these hydrogel compositions are not necessarily physiological. To improve assay capabilities, contractile forces have been measured in 3D muscle bundles with custom force transducers (Davis et al., 2019; Madden et al., 2015; Rao et al., 2018) or by tracking the displacement of pillars (Osaki et al., 2018; Uzel et al., 2016). Similar to 2D tissues, biophysical cues, such as mechanical stretch (Powell et al., 2002) and optogenetic stimulation (Mills et al., 2019), or addition of fibroblasts (Dennis et al., 2001) have also been shown to mature 3D muscle bundles.

Microfluidic devices have also been fabricated to engineer and maintain 3D muscle bundles (Agrawal et al., 2017; Shimizu et al., 2015). These systems are advantageous because they continuously perfuse fresh media to the engineered tissues, which probably improves viability compared to static culture. Furthermore, microfluidic devices can be used to screen drugs at a higher throughput and can be linked to other microfluidic organ-on-chip systems to capture organ-organ interactions, and mimic organism-level responses (Novak et al., 2020).

One common approach to innervate 3D muscle bundles is to directly seed them with spheroids of motor neurons (Afshar Bakooshli et al., 2019; Morimoto et al., 2013; Smith et al., 2016). These systems have demonstrated that the resulting NMJs are functional but still have relatively diffuse acetylcholine receptor clustering (Morimoto et al., 2013). Acetylcholine clustering in 3D muscle bundles has been advanced by adding the basement membrane components agrin and laminin (Wang et al., 2013), which could help improve NMJ formation in these co-cultures. Despite their limited maturity, these 3D neuromuscular tissues show reduced contractility in response to sera from MG patients (Morimoto et al., 2013), recapitulating the pathological response in MG and demonstrating their promise for modeling complex neuromuscular diseases.

Similar to 2D models, compartmentalized microdevices have been developed to culture motor neuron spheroids and engineered muscle bundles in separate compartments connected by axon-permissive channels (Fig. 4) (Osaki et al., 2018, 2020; Uzel et al., 2016). In these studies, the muscle bundles were attached to flexible pillars and the motor neurons were optogenetically modified. With this combination of technologies, the users could quantify muscle contractility as a function of motor neuron stimulation, a key readout of NMJ function. This type of device was also used to capture NMJ degeneration in tissues generated using hiPSC-derived motor neurons from an ALS patient. Importantly, the application of two ALS drug candidates, bosutinib and rapamycin, to this model reduced muscle atrophy and dysfunction (Osaki et al., 2018), demonstrating how this type of approach has the potential for patient-specific disease modeling and drug screening.

## Conclusions

Recently developed approaches to model healthy and diseased neuromuscular tissues on a chip have the potential to capture the vastly heterogeneous genotypes and phenotypes of individuals with a variety of neuromuscular disorders. Newer technologies, such as 3D bioprinting, which is a form of additive manufacturing that uses cells and other biomaterials as ‘inks’ to print living structures (Choi et al., 2016; Costantini et al., 2017b; Kang et al., 2016; Kim et al., 2020), will probably further advance these models. However, *in vitro* models of neuromuscular tissues are far from achieving adult-like maturity, especially when based on hiPSC-derived muscle cells and motor neurons. Furthermore, *in vitro* models of neuromuscular tissues lack the supporting cells known to be important regulators of NMJs in health and disease, such as Schwann cells (Santosa et al., 2018). Most *in vitro* models also currently lack immune cells, despite the established role of neuroinflammation in many neuromuscular diseases (Mäurer et al., 2002; Thonhoff et al., 2018). However, researchers have begun developing models that integrate immune cells, such as macrophages (Juhas et al., 2018), to probe the role of the immune system in muscle injury and repair. Given these limitations, *in vitro* models are most powerful when implemented hand-in-hand with animal models, which have less human relevance but more advanced motor unit structure and physiology. Together, these complementary model systems are likely to pave the way for more effective and personalized therapies for these debilitating diseases.
